# Cyanidin-3-glucoside enhances mitochondrial function and biogenesis in a human hepatocyte cell line

**DOI:** 10.1007/s10616-018-0242-4

**Published:** 2018-08-28

**Authors:** Rashad Mogalli, Toshiya Matsukawa, Osamu Shimomura, Hiroko Isoda, Nobuhiro Ohkohchi

**Affiliations:** 1 Department of Surgery and Organ Transplantation, Faculty of Medicine, University of Tsukuba, Tsukuba, Ibaraki 305-8575 Japan; 2 Graduate School of Life and Environmental Sciences, University of Tsukuba, Tsukuba, Ibaraki 305-8572 Japan; 3 Faculty of Life and Environmental Sciences, University of Tsukuba, Tsukuba, Ibaraki 305-8572 Japan; 4 Alliance for Research on North Africa (ARENA), University of Tsukuba, Tsukuba, Ibaraki 305-8572 Japan

**Keywords:** Cyanidin-3-glucoside, Anthocyanin, Mitochondria, PGC-1α, SIRT1, HuH7

## Abstract

Mitochondrial dysfunction has been identified as one of the primary factors contributing to liver diseases. Pathways that control mitochondrial biogenesis are potential therapeutic targets for the amelioration of hepatocyte dysfunction and liver disease. Research on natural pharmacological agents that ameliorate liver diseases has intensified over the last two decades. Cyanidin-3-glucoside (Cy3g), a dietary flavonoid compound extracted from a wide variety of fruits and vegetables, reportedly has several beneficial health effects. In this study, we used an adult human hepatoma cell line (HuH7) to investigate the effects of the Cy3g polyphenolic compound on mitochondrial function and biogenesis in vitro. An increase in intracellular mitochondrial reductase levels was observed after treatment with Cy3g, but cytotoxicity was not induced. In addition, mitochondrial membrane potential and ATP production were increased following Cy3g treatment. Cy3g treatment also resulted in a dose- and time-dependent upregulation of the gene expression of peroxisome proliferator-activated receptor gamma coactivator 1-alpha (PGC-1α), a transcription factor considered a master regulator of mitochondrial biogenesis and metabolism. Additionally, the expression of sirtuin 1 (SIRT1), which plays a key role in deacetylating PGC-1α, was also increased in a dose- and time-dependent manner. Cy3g treatment also increased the expression of downstream PGC-1α genes, nuclear respiratory factor 1 and mitochondrial transcription factor A (TFAM). Our results suggest that Cy3g has potential as a hepatoprotective therapeutic agent that enhances mitochondrial function and biogenesis in hepatocytes.

**Electronic supplementary material:**

The online version of this article (10.1007/s10616-018-0242-4) contains supplementary material, which is available to authorized users.

## Introduction

Mitochondria are the main organelles that maintain energy production, control metabolism and regulate stress within cells (Wallace 2005; Nunnari and Suomalainen 2012). Mitochondrial health is an important parameter of overall cell health. Mitochondrial dysfunction has been found to be a key factor leading to the development of a variety of diseases, such as neurodegenerative disorders (Johri and Beal 2012), endocrine dysfunction (Chow et al. 2016), and several metabolic disorders, such as insulin resistance (Petersen et al. 2003; Morino et al. 2006), as well as hepatic and cardiovascular diseases (Gustafsson and Gottlieb 2007; Nassir and Ibdah 2014).

Many studies have reported the direct relationship between mitochondrial dysfunction and liver diseases (Pessayre et al. 2002; Begriche et al. 2006; Rector et al. 2010; Nassir and Ibdah 2014). Disrupted hepatocyte metabolism results in lipid retention in hepatocytes. This retention has been attributed to failed long chain fatty acid catabolism via hepatic mitochondrial beta-oxidation (Fabbrini et al. 2010).

In *Homo sapiens* and eukaryotic animals, cell mitochondrial biogenesis and metabolic control are orchestrated by several factors. Among these, one important factor has emerged in the past decade, called Peroxisome proliferator-activated receptor gamma coactivator 1-alpha (PGC-1α). PGC-1α controls mitochondrial biogenesis via the activation of several downstream genes, such as mitochondrial transcription factor A (TFAM), which is triggered by nuclear respiratory factor-1/-2 (NRF1/2) (Finck and Kelly 2006). Fasting or hypothermia, which help the cell adapt to nutritional status, can trigger PGC-1α. Disruption of this mechanism has been associated with the development of mitochondrial dysfunction-related diseases (Vega et al. 2000; Scarpulla 2011). Sirtuin 1 (SIRT1), a homolog of SIRT2, was observed to cooperate with PGC-1α to regulate hepatocyte gluconeogenesis and glycolysis-related genetic controlling processes (Rodgers et al. 2005). A study reported that PGC-1α impairment in the rodent liver results in impaired mitochondrial biogenesis and lipid metabolism, eventually exacerbating fatty liver diseases (Aharoni-Simon et al. 2011).

The search has intensified for natural product treatments that can replace or synergize with current pharmaceutical products and minimize pharmaceutical side effects and financial burdens (Bagchi et al. 2015). Cyanidin-3-glucoside (Cy3g), an anthocyanin dietary flavonoid compound extracted from a wide variety of fruits and vegetables, has been reported to have multiple beneficial effects. A clinical study found that intake of this compound minimizes cardiovascular risk (Cassidy et al. 2013). Cy3g resulted in increased brown adipose tissue mitochondrial function (You et al. 2017). In addition, treating different rodents models with Cy3g resulted in hepatocyte protection and prevented obesity and insulin resistance (Jiang et al. 2014, Wei et al. 2011). Our previous reports indicate that Cy3g benefits skeletal muscle aerobic capacity and enhances adipose tissue metabolism (Matsukawa et al. 2015, 2017).

The HuH7 cell line is a well-differentiated, established adult hepatoma cell line that provides a good in vitro system to test the effects of natural compounds on hepatocyte metabolism (Chavez-Tapia et al. 2012; Krelle et al. 2013). In this study, we investigated the effects of Cy3g on mitochondrial function and biogenesis using the HuH7 cell line as a hepatocyte model.

## Materials and methods

### Chemicals

Cy3g (98% HPLC Purity) was purchased from Tokiwa Phytochemical Co., Ltd. Japan. The well-differentiated human hepatocellular carcinoma HuH7 cell line was purchased from the National Institutes of Biomedical Innovation Health and Nutrition JCRB Bank (JCRB No. JCRB0403, Tokyo, Japan). The cell culture medium was Dulbecco’s modified Eagle’s medium (DMEM) containing low glucose (Sigma, Tokyo, Japan). Penicillin/streptomycin and trypsin/EDTA were obtained from Lonza (Tokyo, Japan). Fetal bovine serum (FBS) and Hanks’ balanced salt solution (HBSS) were purchased from Gibco (USA). Sodium dodecyl sulfate (SDS) was purchased from Wako (Tokyo, Japan). 4-(2-Hydroxyethyl)-1-piperazineethanesulfonic acid (HEPES) and Triton X-100 were purchased from Sigma (MO, USA). MTT was purchased from Dojindo Co., Ltd. (Kumamoto, Japan). Guava ViaCount and Check Kit reagents were purchased from Guava Technologies Co., Ltd. USA. Rhodamine 123 was purchased from Wako (Tokyo, Japan).

### Cell culture

Cells were cultured in 75-cm^2^ culture flasks with low glucose DMEM supplemented with 10% heat-inactivated FBS and 1% penicillin/streptomycin (5000 IU/ml/5000 μg/ml) at 37 °C in an incubator with 5% CO_2_. The growth medium was changed every other day, and the experiments were completed with cells between passages 3–7 and at no more than 70–80% confluence. Passaging was performed with trypsin/EDTA.

### MTT assay

Cells were seeded at a concentration of 3 × 10^4^ cells per well in DMEM 10% FBS culture medium in 96-well plates and cultured for 24 h. Then, the cells were treated with different concentrations of Cy3g compound for different time intervals. Under dark settings, the cells were then washed with phosphate-buffered saline (PBS) and incubated with MTT reagent (5 g/l) for 3 h. MTT formazan crystals were then dissolved in 10% SDS and kept overnight at room temperature. Optical density was measured using a Powerscan HT plate reader (Dainippon Sumitomo Pharma Co., Ltd., Japan). The results were normalized to those of the control group. All experiments were performed in triplicate.

### Guava cell count

The Guava ViaCount assay was conducted on untreated and treated suspension cultures according to the manufacturer’s protocol (Cat. No. 4000-0040). Guava ViaCount staining reagent (380 µl) was added and mixed with 20 µL of cell suspension in a 1.5-mL tube. Then, the suspension was kept in the dark for 5 min at room temperature. A Guava PCA machine (Guava Technologies) was used to analyze the samples. Readings were acquired using Cytosoft software (version 2.1.2). Machine performance was assessed using the Guava check application with a Guava Check kit (Cat. No. 4500-0020).

### ATP assay

Cellno ATP assay reagent (Toyo Inc., Tokyo, Japan) was used in accordance with the manufacturer’s protocol to measure intracellular ATP levels. HuH7 cells were treated with different concentrations of Cy3g compound for various times and then incubated with ATP assay reagents for 15 min at room temperature. A Powerscan HT plate reader (Dainippon Sumitomo Pharma Co., Ltd.) was used to detect luminescence.

### Mitochondrial membrane potential (MMP)

MMP was measured using rhodamine 123 fluorescent dye. Cells were treated with Cy3g at various concentrations and time intervals. Then, the cells were incubated with the rhodamine 123 dye (10 µg/ml) in 10 mM HEPES-HBSS buffer (pH 7.4) for 20 min at 37 °C. After lysing HuH7 cells using 1% Triton X-100 (Sigma-Aldrich™ Co., Ltd., USA), a Powerscan HT plate reader (Dainippon Sumitomo Pharma Co., Ltd., Japan) was used to quantify rhodamine 123 fluorescence intensity (excitation 485 nm/emission 528 nm).

### Real-time PCR analysis

HuH7 cells were plated in a 60-mm cell culture dish. After 24 h, the cells were incubated with different Cy3g concentrations for different times (1, 3, 6, and 24 h). Total RNA from HuH7 cells was isolated using Macherey–Nagel’s RNA extraction kit (Macherey–Nagel GmbH & Co. KG, Germany). The extraction process was executed according to the manufacturer’s protocol. The quantity of RNA was evaluated using a NanoDrop 2000 Spectrophotometer (Thermo Scientific™ Co., Ltd., USA). Reverse transcription (RT) reactions were carried out with the SuperScript III RT kit (Invitrogen Co., Ltd., Carlsbad, CA, USA). The following primer sets and TaqMan probes for experimental genes were purchased from Applied Biosystems (CA, USA): GADPH (Hs02786624_g1), PGC-1α (Hs01016719_m1), TFAM (Hs00273327_s1), NRF1 (Hs00602161_m1), SIRT1 (Mm00490758_m1), CPT-1β (Hs03046298_s1) and PFK1 (Hs01075411_m1). The mRNA expression level of each gene was normalized using GADPH as an internal control.

### Statistical analysis

All experiments were repeated three times. The experimental data are presented as the mean ± standard deviation. Microsoft Excel (iOS Version 2011; Microsoft Inc., USA). When two values were compared (control vs. treatment), statistical significance was assessed using Student’s unpaired *t* test. *P* ≤ 0.05 was considered statistically significant.

## Results

### Cy3g increased mitochondrial number and function without inducing cytotoxicity or proliferation

**Fig. 1:**
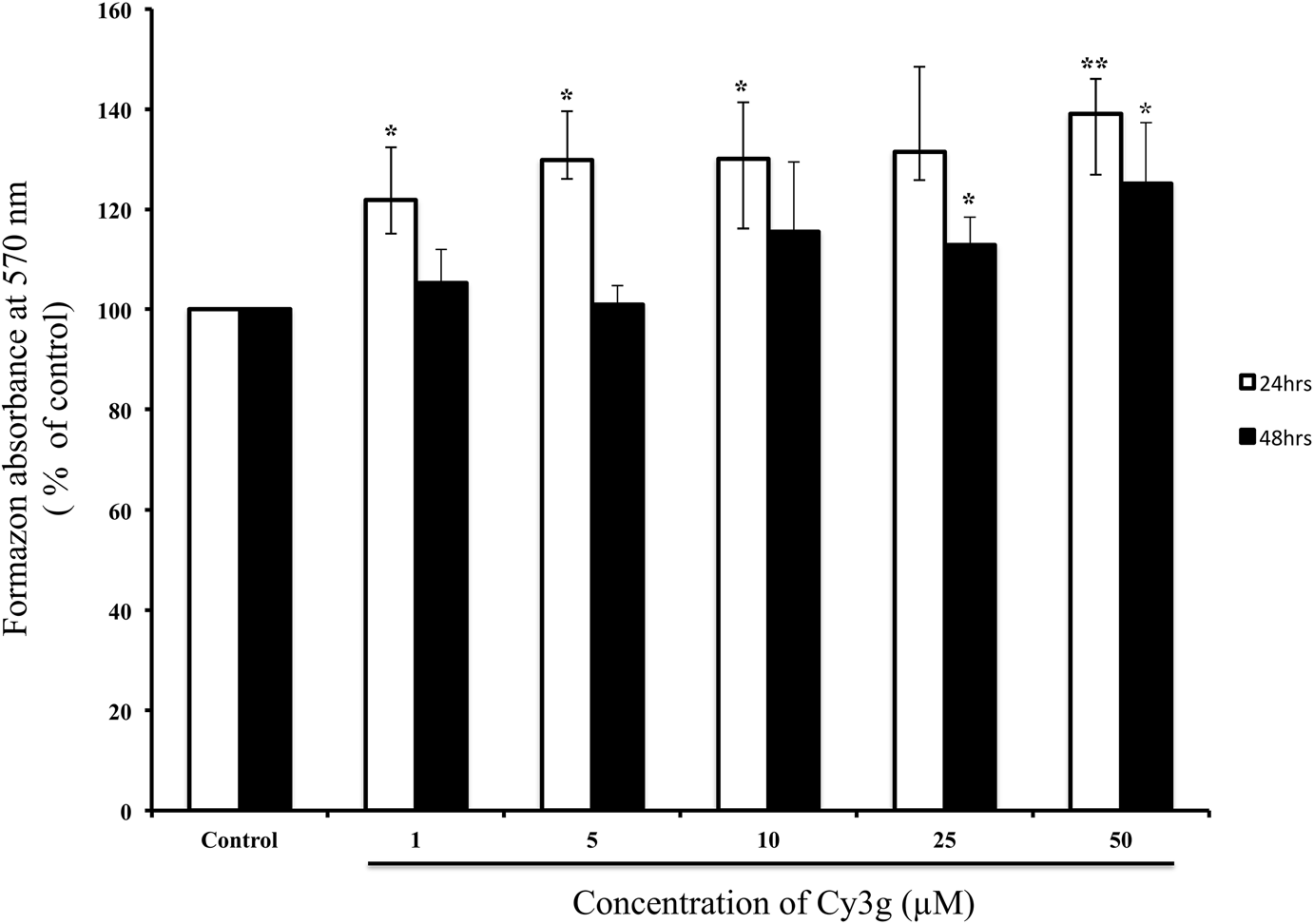
Cell proliferation was measured using MTT assays, and the HuH7 adult human hepatoma cell line was cultured at 3 × 10^4^ cells/mL per well with or without (control) different concentrations of Cy3g (1, 5, 10, 25 or 50 µM) for different time periods (24 or 48 h). Values represent the means of three independent experiments ± standard deviation. Bars with asterisks are significantly different from the control at *P* ≤ 0.05 (*) or *P* ≤ 0.01 (**)

The MTT (3-(4,5-dimethylthiazol-2-yl)-diphenyl tetrazolium bromide) assay was used to examine the cytotoxicity of the Cy3g compound on the HuH7 hepatoma cell line at different times and concentrations. The study revealed that Cy3g did not reduce cell viability. The MTT assay is a classic method for evaluating cell mitochondrial function via intracellular mitochondrial reduction of MTT to formazan (Brand and Nicholls 2011). Compared with the control, the increased absorbance induced by 25 and 50 µM Cy3g indicated that cell mitochondrial activity was upregulated dose-dependently by 130 and 139%, respectively, at 24 h. In addition, absorbance was increased 112 and 125% after treatment with 25 and 50 µM Cy3g, respectively, at 48 h (Fig. 1). Guava cell counting was used to assess cell proliferation: treatment with Cy3g did not decrease or increase cell proliferation (Supplementary Fig. 1).

### Cy3g increased intracellular ATP production in HuH7 cells

**Fig. 2:**
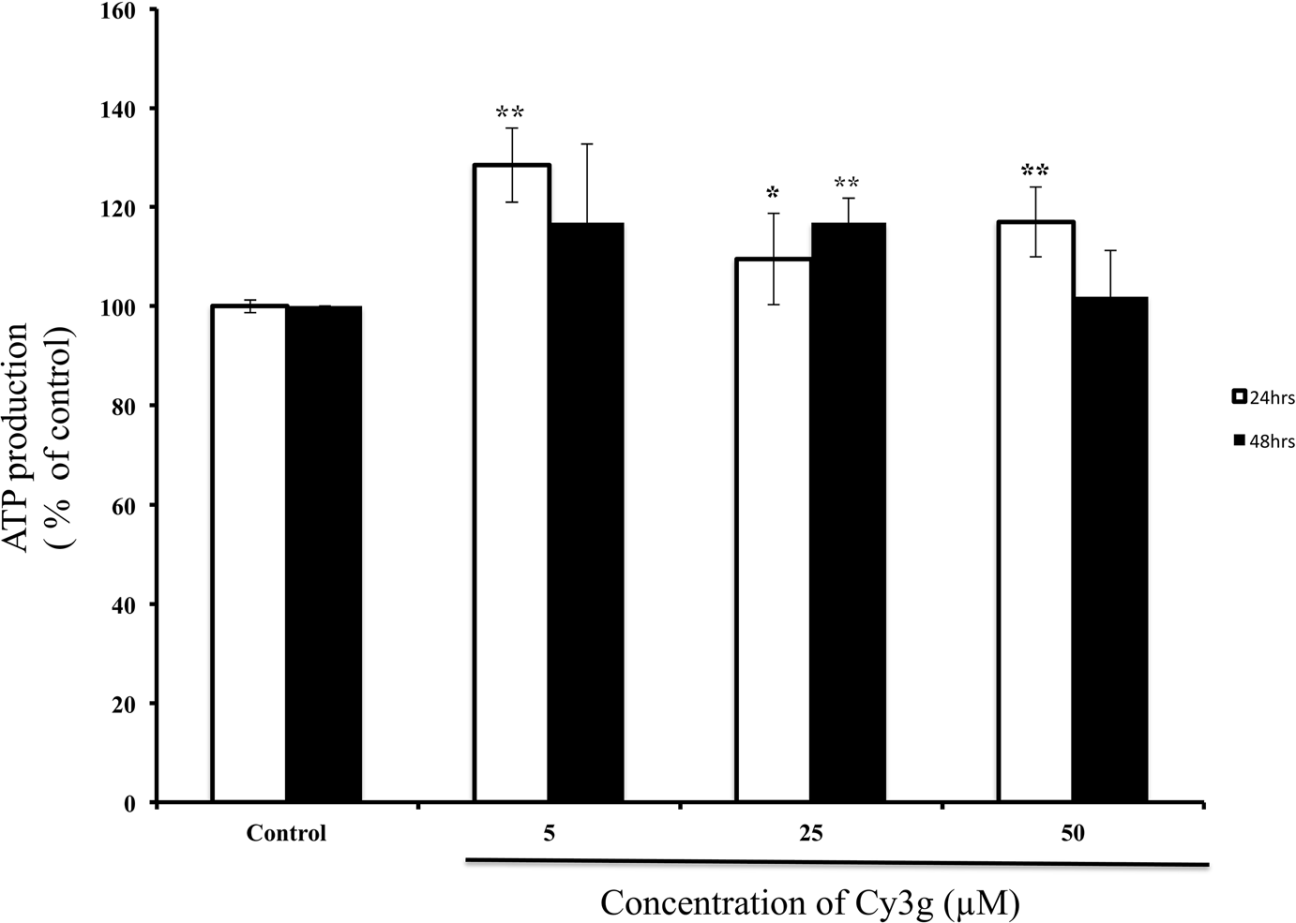
Measurement of intracellular ATP in the HuH7 adult human hepatoma cell line cultured without Cy3g (control) or with different concentrations of Cy3g (5, 25 or 50 µM) for different time periods (24 or 48 h). Values represent the means of three independent experiments ± standard deviation. Bars with asterisks are significantly different from the control at *P* ≤ 0.05 (*) or *P* ≤ 0.01 (**)

MTT assay was used to determine the effects of Cy3g-induced mitochondrial content on the intracellular ATP levels in HuH7 cells. However, the compound had no cytotoxic effect on HuH7 cells (MTT assay result). Luminescence readings indicated that compared with the control treatment, Cy3g treatment significantly increased the mitochondrial ATP level to 109% at the 25 µM concentration and to 117% at the 50 µM concentration (Fig. 2).

### Cy3g increased the MMP

**Fig. 3:**
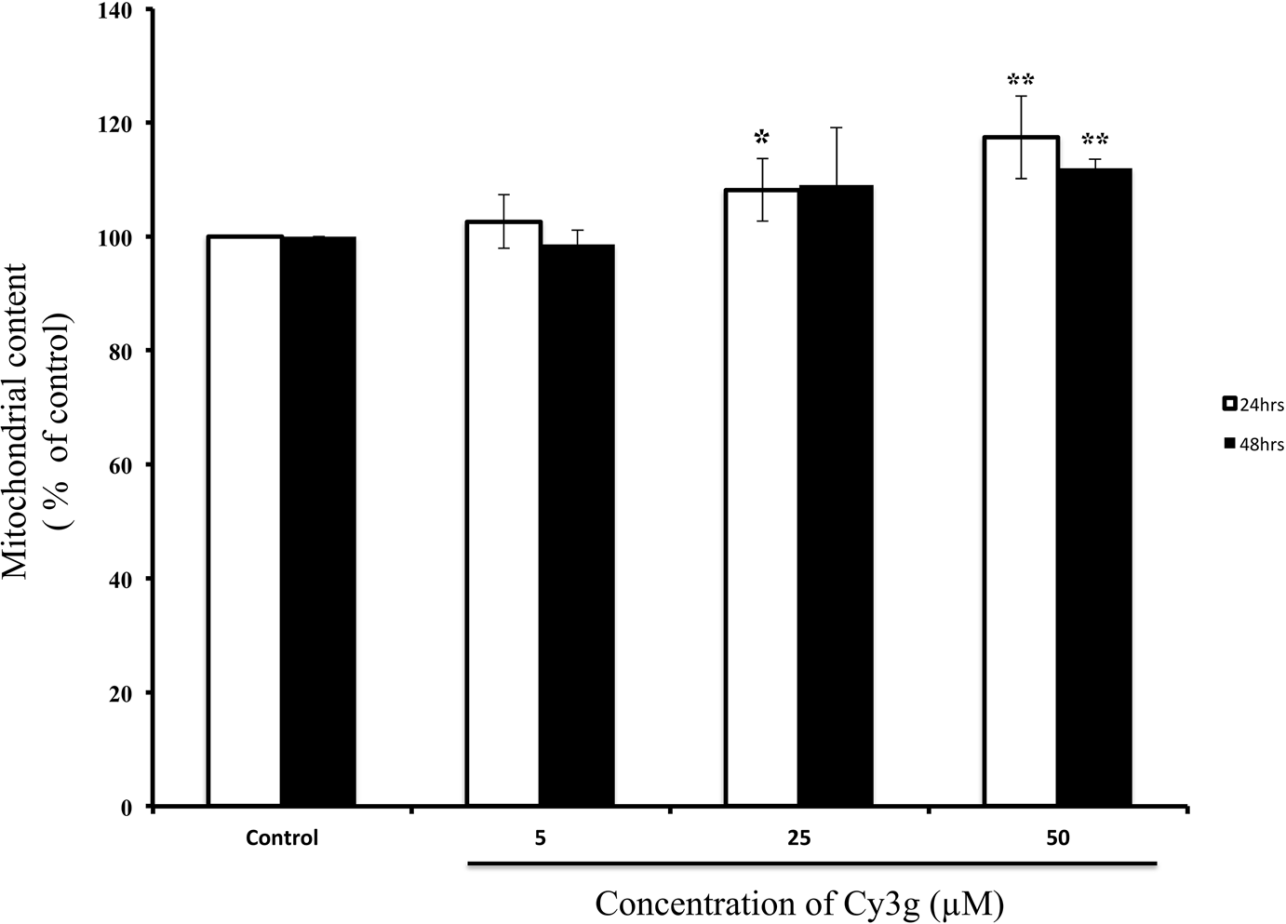
Measurement of the mitochondrial content in the HuH7 adult human hepatoma cell line cultured without Cy3g (control) or with different concentrations of Cy3g (5, 25 or 50 µM) for different time periods (24 or 48 h). Values represent the means of three independent experiments ± standard deviation. Bars with asterisks are significantly different from the control at *P* ≤ 0.05 (*) or *P* ≤ 0.01 (**)

The majority of ATP production occurs within the mitochondria via oxidative phosphorylation: the mitochondrial electron transport chain creates an electrochemical gradient that induces ATP synthesis and generates the MMP, which is a good indicator of mitochondrial health (Sakamuru et al. 2016). Rhodamine 123 is a cationic, membrane-permeable fluorescent staining dye that is used to measure the inner MMP), which is a sensitive indicator of mitochondrial health (Sakamuru et al. 2016). In our experiment, compared with those of control-treated cells after 24 h, fluorescence readings significantly increased to 108 and 117% in 25 and 50 µM Cy3g-treated cells, respectively. After 48 h of treatment, fluorescence was increased 112% in cells treated with 50 µM Cy3g (Fig. 3), indicating that Cy3g increased the mitochondrial content in HuH7 cells.

### Cy3g treatment dose- and time-dependently increased PGC-1α and SIRT1 gene expression levels

**Fig. 4:**
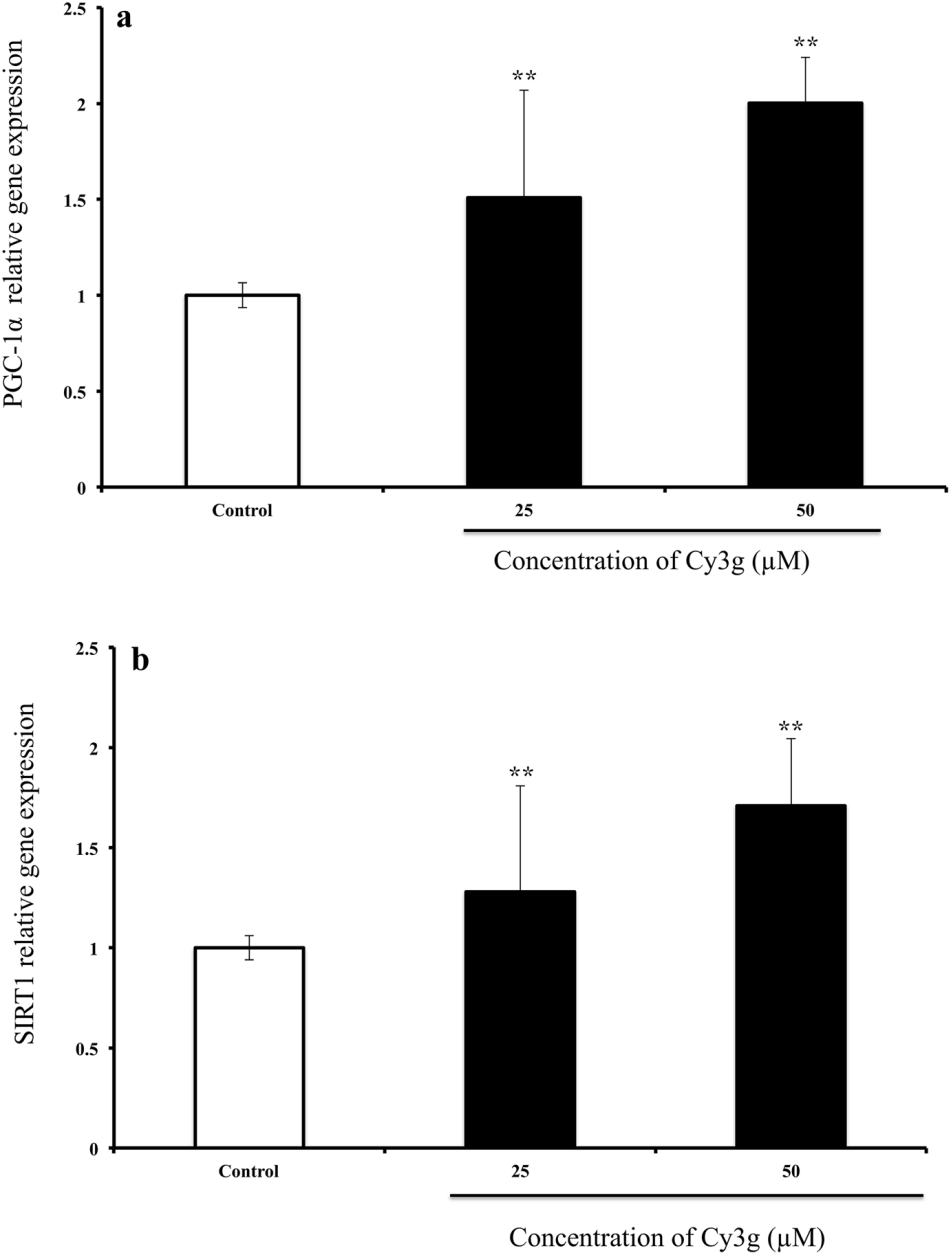
Effects of Cy3g on HuH7 cell PGC-1α (**a**) and SIRT1 (**b**) mRNA expression levels after treatment with different concentrations of Cy3g (25 and 50 µM) for 24 h; the gene expression level was normalized to the GADPH expression level. Values are expressed as the mean ± S.E.M. of triplicate experiments. *P ≤ 0.05 and **P ≤ 0.01 indicate that the mean value is significantly different from that of the control group

The polyphenol Cy3g dose-dependently upregulated PGC-1α and SIRT1 gene expression, and these genes have been reported to play a key role in the control of metabolic adaption and mitochondrial biogenesis (Finck and Kelly 2006; Ventura-Clapier et al. 2008; Cantó and Auwerx 2009). Cy3g significantly upregulated PGC-1α gene expression in a dose-dependent manner, from a 1.5-fold change at the 25 µM dose to a twofold change at the 50 µM dose (*P ≤ 0.01) (Fig. 4a). Regarding SIRT1 gene expression, 25 and 50 µM Cy3g increased the level 1.2- to 1.7-fold, respectively (Fig. 4b).

**Fig. 5:**
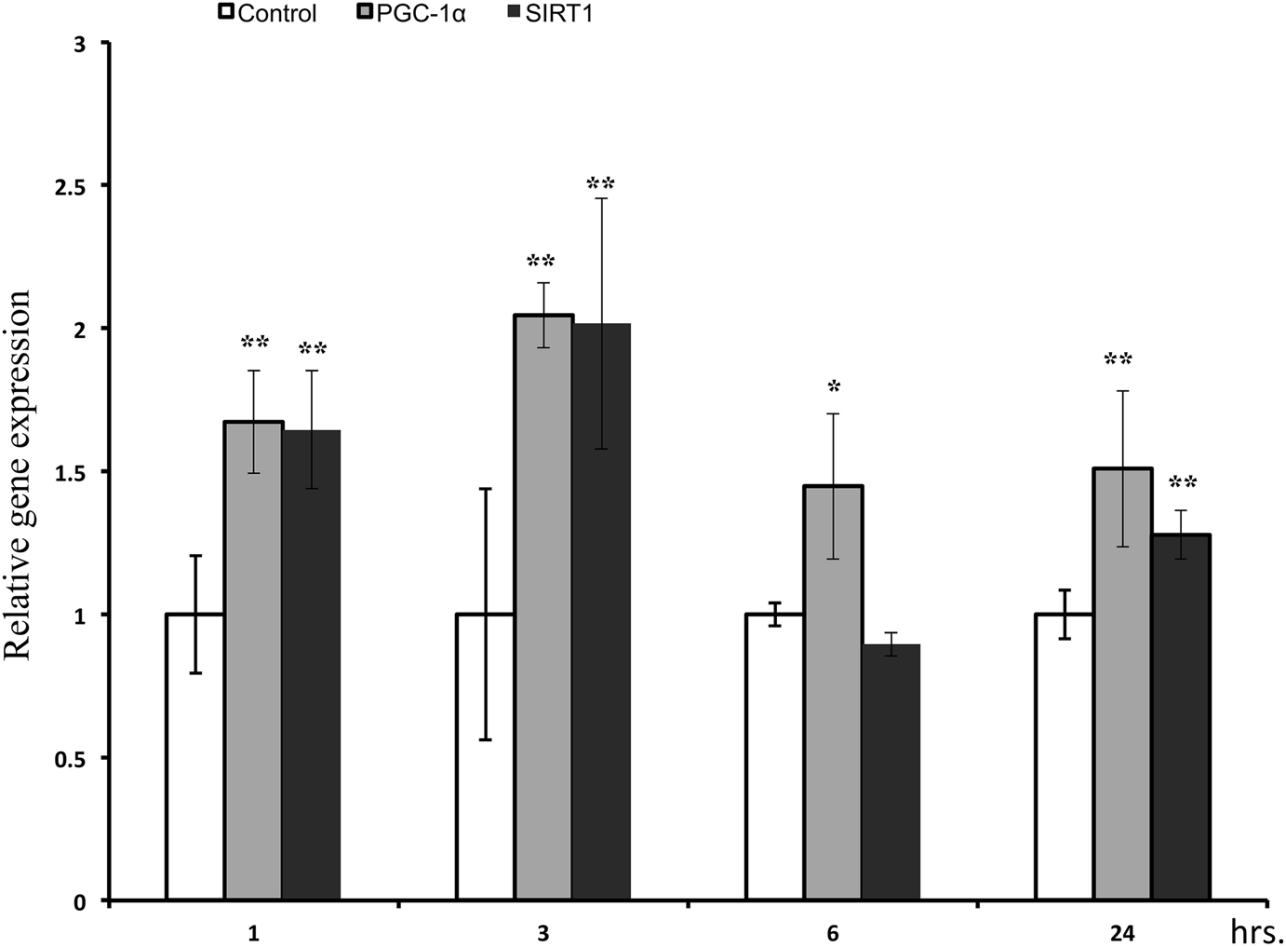
Effects of Cy3g on HuH7 cell PGC-1α and SIRT1 mRNA expression levels were measured at different time intervals (1, 3, 6 and 24 h after treatment with Cy3g), and the gene expression level was normalized to the GADPH expression level. Values are expressed as the mean ± S.E.M. of triplicate experiments. *P ≤ 0.05 and **P ≤ 0.01 indicate that the mean value is significantly different from that of the control group

Cy3g resulted in a time-dependent increase in PGC-1α and SIRT1 gene expression levels. The 25 µM concentration was selected to evaluate SIRT1 and PGC-1α levels at different time intervals (1, 3, 6, and 24 h). PGC-1α and SIRT1 gene expression levels peaked at 3 h in treated cells compared with those in control cells, with significant 2.0-fold increases (*P ≤ 0.01). Cy3g treatment also significantly increased SIRT1 and PGC-1α gene expression after 24 h, with fold changes of 1.50 and 1.27, respectively (*P* ≤ 0.01) (Fig. 5).

### The effect of Cy3g on PGC-1α and SIRT1 downstream genes

**Fig. 6:**
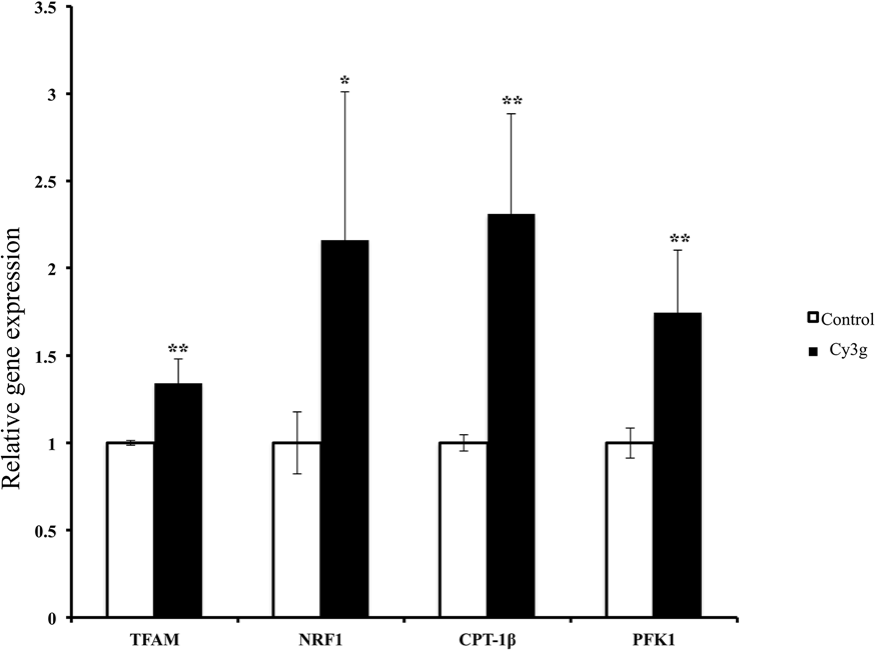
Effects of Cy3g on HuH7 cell PGC-1α downstream gene mRNA expression levels were measured at 24 h. After treatment with 25 µM Cy3g, the gene expression levels (TFAM, NRF1, CPT-1β, and PFK-1) were normalized to the GADPH expression level. Values are expressed as the mean ± S.E.M. of triplicate experiments. *P ≤ 0.05 and **P ≤ 0.01 indicate that the mean value is significantly different from that of the control group

Treatment with Cy3g (25 µM) for 24 h significantly increased the expression of PGC-1α-coactivated downstream genes, such as NRF1, approximately 2.5-fold. Cy3g increased the TFAM level by approximately 1.3-fold. TFAM is a nuclear-encoded transcription factor that plays a key role in mitochondrial DNA replication and transcription and is regulated by NRF1 (Finck and Kelly 2006). In addition, Cy3g increased CPT-1β levels approximately 2.3-fold (Fig. 6). CPT-1β is located within the mitochondrial outer membrane and is considered the rate-limiting enzyme of mitochondrial β-oxidation, as it controls mitochondrial uptake of long chain acyl-CoA fatty acids; increased PGC-1α levels have been found to increase the CPT-1β level, thus increasing the fatty acid oxidative capacity of the mitochondria (Song et al. 2010; Nikolić et al. 2012). Furthermore, Cy3g treatment increased phosphofructokinase 1 (PFK-1) gene expression approximately 1.7-fold. PFK-1 is an important regulator of glycolysis (Han et al. 2016) (Fig. 6).

## Discussion and conclusion

The search for natural herbal compounds and extracts that treat or prevent disease has intensified during the last decade. Several studies have reported the beneficial effects of a variety of natural compounds, such as resveratrol, quercetin and catechin on health (Watson et al. 2013).

Among anthocyanin compounds, Cy3g, a phenol pigment that belongs to the flavonoid family, has been shown to have beneficial effects in several in vitro and in vivo clinical trials (Watson et al. 2013). Cy3g enhances skeletal muscle mitochondrial biogenesis by upregulating PGC-1α levels (Matsukawa et al. 2017). PGC-1α is reportedly an essential factor for upregulating hepatic metabolism and is key for overall liver metabolism (Leone et al. 2005; Finck and Kelly 2006). This study examined the ability of Cy3g to increase mitochondrial function and biogenesis in hepatic cells (HuH7) and elucidated the underlying mechanism. In this study, we found that Cy3g induced PGC-1α activity. This induction of PGC-1α gene expression was associated with a similar tendency for increased SIRT1 gene expression.

Increased expression of PGC-1α-coactivated downstream genes, such as nuclear respiratory factor-1 (NRF1), which encodes respiratory chain subunits and other proteins necessary for mitochondrial function, was observed (Finck and Kelly 2006). Moreover, Cy3g increased the gene expression of mitochondrial transcription factor A (TFAM), a nuclear-encoded transcription factor that plays a key role in mitochondrial DNA replication and transcription and is regulated by NRF1 (Finck and Kelly 2006). Cy3g also increased CPT-1β gene expression, which is located within the mitochondrial outer membrane and is considered the rate-limiting enzyme of mitochondrial β-oxidation as CPT-1β controls the mitochondrial uptake of long chain acyl-CoA fatty acids; increased PGC-1α levels have been found to increase the CPT-1 β level, thus increasing the fatty acid oxidative capacity of mitochondria (Song et al. 2010; Nikolić et al. 2012). Cy3g also increased PFK-1 gene expression, an important regulator of glycolysis (Han et al. 2016).

Currently, lifestyle modification and caloric restriction are the only treatments for nonalcoholic fatty liver diseases (Nassir and Ibdah 2016). Some polyphenol compounds, such as resveratrol, have shown calorie restriction-mimicking effects in mammalian diseases and can ameliorate liver fat accumulation in high-fat diet mouse models, mostly due to the activation of metabolism-sensing signaling systems (Baur et al. 2006; Ajmo et al. 2008; Li et al. 2016).

While several pathways control mitochondrial function, biogenesis and free fatty acid oxidation, a recently identified member of the Peroxisome proliferator-activated receptor gamma (PPAR-γ) coactivator family, PGC-1α, serves as a major regulator of the nuclear receptors that control metabolic pathways and is expressed in tissues with high oxidative capacity (Finck and Kelly 2006). Hepatocytes extracted from PGC-1α-deficient mice exhibit reduced mitochondrial respiration rates, indicating a reduced hepatic fatty acid oxidation capacity (Leone et al. 2005). SIRT1 coexists with the transcription factor PGC-1α and plays an important role in PGC-1α activation via deacetylation (Rodgers et al. 2005). SIRT1 and PGC-1α signaling is important in the protection of in vitro hepatocyte models against mitochondrial oxidative stress (Tan et al. 2015). Furthermore, pharmacological activation of SIRT1 by polyphenol in HepG2 protected against FAS induction and lipid accumulation (Hou et al. 2008).

Several studies have revealed the crucial role of sirtuins generally and SIRT1 specifically in liver diseases (Nassir and Ibdah 2016; Ding et al. 2017). SIRT1, an NAD + -dependent protein deacetylase, is an important regulator of energy homeostasis, enhanced mitochondrial metabolism, antioxidative protection, lipid catabolism and glucose homeostasis (Canto and Auwerx 2012). Both in vitro and in vivo models of SIRT1 deficiency have shown a tendency for increased lipid accumulation in the liver and downregulation of de novo hepatic lipid synthesis transcription factors, such as sterol regulatory element binding protein-1c (SREBP-1c) and carbohydrate response element binding protein (ChREBP) (Purushotham et al. 2009; Wang et al. 2010). Previously, Guo et al. (2012) reported that Cy3g decreased lipid accumulation in hepatocytes. Jiang et al. (2014) demonstrated the beneficial effect of Cy3g in protecting primary mouse hepatocytes from hyperglycemia-induced mitochondrial depolarization, and preincubation with Cy3g improved cell survival and reduced reactive oxygen species (ROS) generation by modulating mitochondrial dysfunction. Pathways that control mitochondrial biogenesis have been studied extensively to identify future therapeutic approaches to treat the mitochondrial dysfunction that leads to various liver and metabolic diseases (Davinelli et al. 2013).

Our experiments showed that in a human-derived hepatocyte model (HuH7), Cy3g is a potent activator of the SIRT1 and PGC-1α signaling pathways, inducing mitochondrial biogenesis and function and triggering an increase in PGC-1α downstream genes, and these effects are dose- and time-dependent. Therefore, this compound should be considered a therapeutic or preventive approach for diseases caused by hepatic cell mitochondrial dysfunction.

## Electronic supplementary material

Below is the link to the electronic supplementary material.

Supplementary material 1 (JPG 1115 kb)

## Abbreviations

Cy3g: Cyanidin-3-glucoside
PGC-1α: Peroxisome proliferator-activated receptor gamma coactivator 1-alpha
SIRT1: Sirtuin 1
MMP: Mitochondrial membrane potential
ATP: Adenosine triphosphate
TFAM: Mitochondrial transcription factor A
NRF1: Nuclear respiratory factor-1
CPT-1β: Carnitine palmitoyltransferase 1 beta
PFK-1: Phosphofructokinase 1
