# Supplementary figures and images for: Cyanidin-3-glucoside enhances mitochondrial function and biogenesis in a human hepatocyte cell line
Source: Cytotechnology. 2018 Aug 28;70(6):1519–28. doi: 10.1007/s10616-018-0242-4 (PMC6269359; doi:10.1007/s10616-018-0242-4)

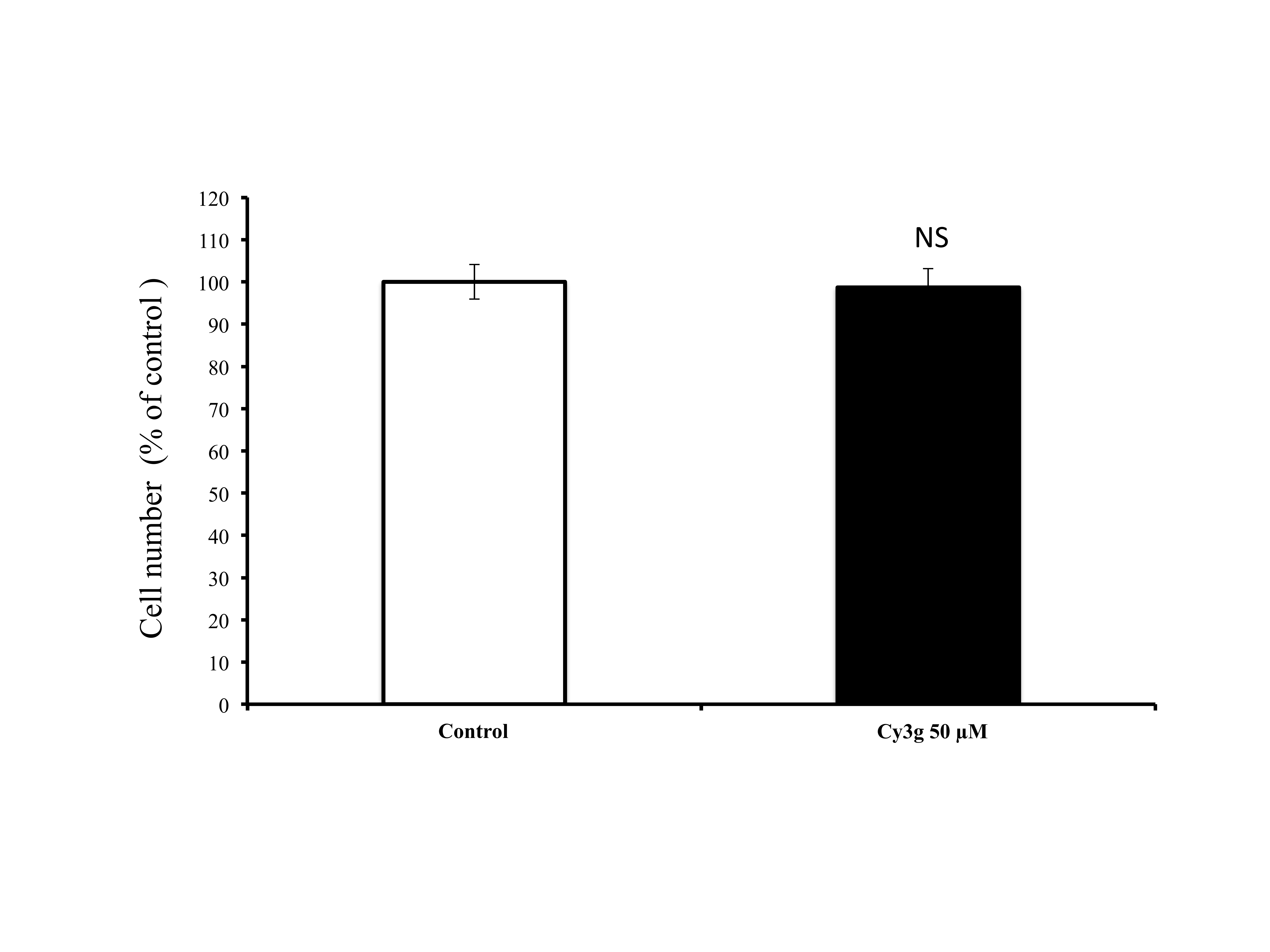

Supplement: Supplementary file 1 — Supplementary material 1 (JPG 1115 kb) [file 10616_2018_242_MOESM1_ESM.jpg]
